# Frequency of Natural Resistance within NS5a Replication Complex Domain in Hepatitis C Genotypes 1a, 1b: Possible Implication of Subtype-Specific Resistance Selection in Multiple Direct Acting Antivirals Drugs Combination Treatment

**DOI:** 10.3390/v8040091

**Published:** 2016-03-25

**Authors:** Sabrina Bagaglio, Andrea Andolina, Marco Merli, Caterina Uberti-Foppa, Giulia Morsica

**Affiliations:** 1 Infectious Diseases Department, Scientific Institute Ospedale San Raffaele, 20127 Milan, Italy; andolinaandrea9@gmail.com (A.A.); merli.marco@hsr.it (M.M.); uberti.caterina@hsr.it (C.U.-F.); morsica.giulia@hsr.it (G.M.); 2 Vita-Salute University, 20132 Milan, Italy

**Keywords:** HCV natural resistance, HCV genotypes, NS5a inhibitors

## Abstract

Different HCV subtypes may naturally harbor different resistance selection to anti-NS5a inhibitors. 2761 sequences retrieved from the Los Alamos HCV database were analyzed in the NS5a domain 1, the target of NS5a inhibitors. The NS5a resistance-associated polymorphisms (RAPs) were more frequently detected in HCV G1b compared to G1a. The prevalence of polymorphisms associated with cross-resistance to compounds in clinical use (daclatasvir, DCV, ledipasvir, LDV, ombitasvir, and OMV) or scheduled to come into clinical use in the near future (IDX719, elbasvir, and ELV) was higher in G1b compared to G1a (37/1552 (2.4%) in 1b sequences and 15/1209 (1.2%) in 1a isolates, *p* = 0.040). Interestingly, on the basis of the genotype-specific resistance pattern, 95 (6.1%) G1b sequences had L31M RAP to DCV/IDX719, while 6 sequences of G1a (0.5%) harbored L31M RAP, conferring resistance to DCV/LDV/IDX719/ELV (*p* < 0.0001). Finally, 28 (2.3%) G1a and none of G1b isolates harbored M28V RAP to OMV (*p* < 0.0001). In conclusion, the pattern of subtype-specific resistance selection in the naturally occurring strains may guide the treatment option in association with direct acting antivirals (DAAs) targeting different regions, particularly in patients that are difficult to cure, such as those with advanced liver disease or individuals who have failed previous DAAs.

## 1. Introduction

The optimal combination of direct acting antivirals (DAAs) has yet to be determined. The class of NS5a inhibitors has pico-molar potency activity, and recent clinical studies have shown these inhibitors to be an important component of DAA combination regimens.

One difficulty in determining the components of an optimal anti-HCV regimen is identifying the DAA agents/classes that are most critical to the overall effectiveness of the combination regimen and can act as a scaffold to which other DAA classes are added.

NS5a inhibitors have no known cross-resistance with other DAA classes, making them ideal agents to use in patients who have failed previous therapies, including those containing DAA agents. It should be noted that several NS5a mutations have been identified in patients naive to treatment with DAAs, which could significantly reduce the antiviral activity of NS5a inhibitors. Some of these baseline polymorphisms could predict virological failure in DAA combination regimens. Within HCV therapy, it should be noted that, although baseline NS5a resistance-associated variants have been overcome in combination regimens, the emergence of treatment-associated NS5a resistance-associated variants has been observed in patients who experience relapse after treatment with an NS5a-containing combination regimen [1,2,3,4,5,6,7,8]. Although the full clinical effect of treatment-associated NS5a resistant variants needs to be investigated in larger studies of this relatively rare, relapsing population, the data highlight the importance of identifying these polymorphisms, especially in the context of different resistance pattern to different NS5a inhibitors.

## 2. Materials and Methods

### 2.1. Study Population

The present study included 2761 NS5a domain1 sequences (amino acids, aa 1–100) deposited before 2010 into the Los Alamos HCV database [9] and derived from NS5a inhibitors-naive patients infected with the HCV genotype (G)1a (*n* = 1209) and G1b (*n* = 1552). To ensure the quality of the data, public sequences were excluded from the analysis if they contained stop codons in NS5a domain 1. Multiple sequences from the same patients and recombinant or clonal sequences were excluded from the analysis. According to the Los Alamos database indications, geographical origin was defined in 613 G1a isolates: 71 sequences were from Europe, 534 sequences from USA, and the remaining 8 from Asia; among 966 G1b isolates, 525 sequences were from Europe, 210 sequences from USA, and 231 sequences from Asia. For 596 G1a and 586 G1b isolates, the geographical origin was not available.

### 2.2. Analysis of Resistance Associated Polymorphisms (RAPs)

To analyze the prevalence of RAPs among these 2761 HCV sequences, a total of 6 positions related to 13 substitutions causing drug resistance to NS5a inhibitors in clinical use (daclatasvir, DCV, ledipasvir, LDV, ombitasvir, and OMV) or scheduled to come into clinical use in the near future (IDX719, elbasvir, and ELV) were considered.

**Figure 1:**
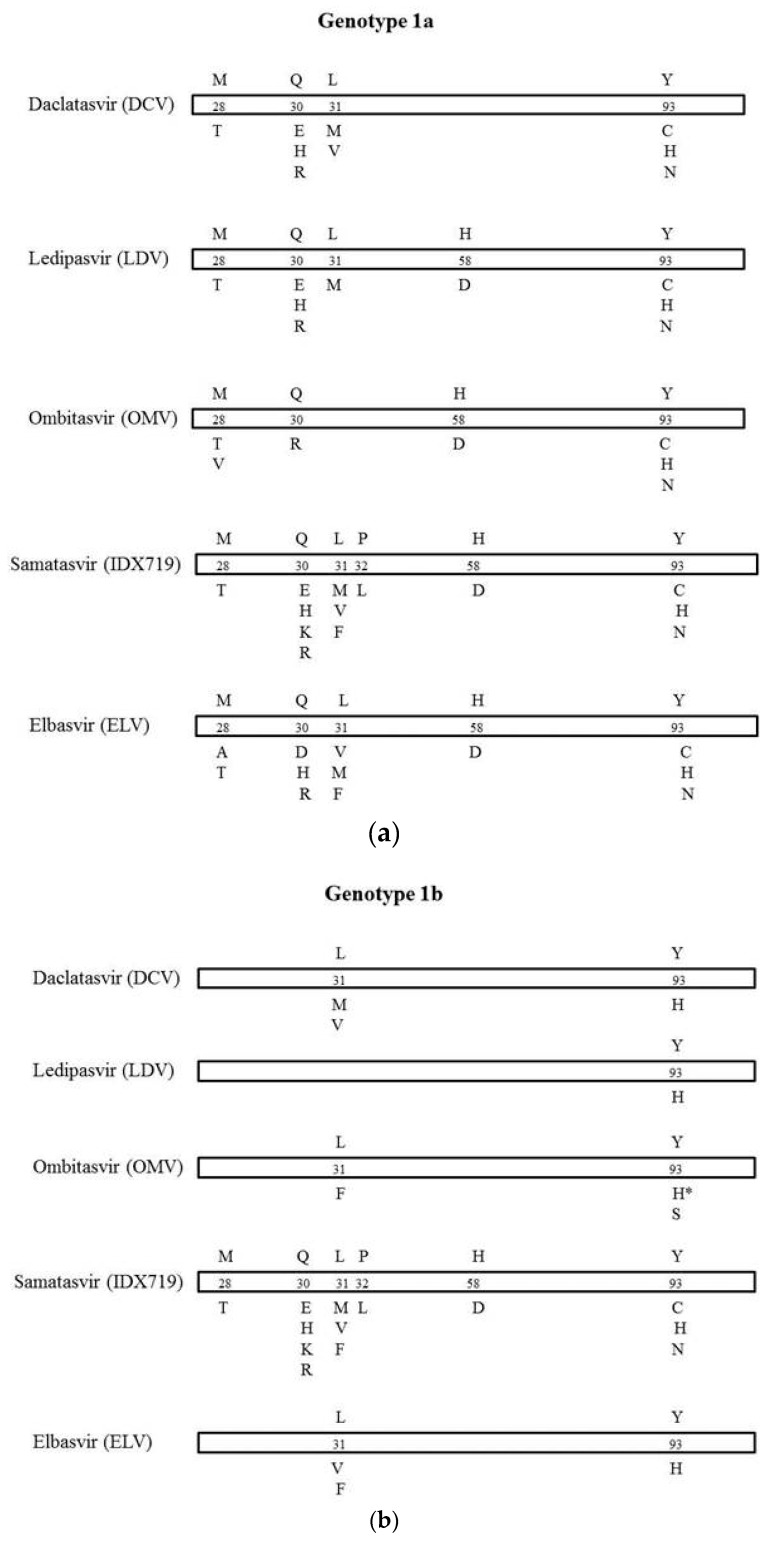
Amino acid substitutions in HCV NS5a domain1 associated with resistance to NS5a inhibitors in clinical use (DCV, LDV, and OMV) or scheduled to come in the near future (IDX719 and ELV) in G1a (**a**) and in G1b (**b**).

The natural mutational profile of the NS5a domain1 was analyzed via multiple alignments of deduced aa sequences. RAPs to DCV, LDV, and OMV were identified according to Lontok *et al*. [10] and to Krishnan *et al.* [11]. In detail, clinically relevant mutations detected in treated patients who experienced on or post-treatment virological failure in completed phase 2 and phase 3 trials were considered. RAPs to IDX719 and ELV were identified *in vitro* according to Bilello *et al*. [12] and Liu *et al*. [13] (Figure 1).

The number in the bar represents the amino acid position. The letter above the number refers to the wild-type amino acid, and the letter(s) below the bars represents the resistance substitutions. For the drug with an asterisk, reported substitutions are associated with on- or post-treatment virological failure, and fold change was not measured *in vitro*.

NS5a mutational pattern was confirmed by Geno2Pheno algorithm. Isolate H77 (GenBank accession number AF009606) and isolate HCV-J (GenBank accession number D90208) were used as G1a and genotype G1b reference sequence, respectively.

Additionally, 10 sequences belonging to G1a that harbored 2 aa substitutions not previously described in clinical trials were classified as double resistant mutants. aa substitutions with >2 fold changes were considered. RAPs to DCV, LDV, and OMV fold changes were obtained from Lontok *et al*. [10] and Krishnan *et al.* [11]. RAPs to IDX719 fold changes were obtained from Bilello *et al*. [12], and RAPs to ELV fold changes were obtained from Liu *et al*. [13] (Table 1).

### 2.3. Statistical Analysis

The frequencies of RAPs were compared using a chi-square test. A *p*-value of <0.05 was considered statistically significant.

## 3. Results

In the present study, RAPs to NS5a inhibitors were detected in 206/2761 (7.5%) G1 isolates retrieved from the Los Alamos database.

RAPs were detected in 69/1209 (5.7%) G1a sequences and in 137/1552 (8.8%) G1b isolates (*p* < 0.0001). Single RAP was revealed in 59/1209 (4.9%) G1a and in 133/1552 (8.6%) G1b isolates (*p* < 0.0001). HCV isolates with at least 2 RAPs were found in 10/69 (14.5%) G1a mutated sequences and in 4/137 (3%) G1b mutated sequences (*p* = 0.0057). The RAP more frequently observed in G1a was M28V (2.3%). In G1b isolates, L31M (6.1%) or Y93H (2.4%) was most commonly detected.

The specific RAPs detected in G1a and G1b isolates are summarized in Table 1.

In regard to genotype-specific cross-resistance, 15 (1.2%) G1a isolates showed a profile of resistance to all NS5a inhibitors considered: DCV, LDV, OMV, IDX719 and ELV; 13 (1%) sequences showed RAPs to DCV/LDV/IDX719/ELV, and 3 (0.25%) isolates were resistant to LDV/OMV/ELV. In G1b, 37 (2.4%) isolates showed cross-resistance to all NS5a inhibitors considered: DCV, LDV, OMV, IDX719, and ELV; 95 (6.1%) isolates showed cross-resistance to DCV and IDX719, and only one isolate harboring L31F substitution showed cross-resistance to OMV/IDX719/ELV. None of the G1b isolates showed L31F plus A92E double mutant or L28M plus R30Q plus Y93H triple mutant. Comparison of the cross-resistance pattern between subtype 1a and 1b showed a higher cross-resistance profile in G1b than in G1a (*p* = 0.040).

Interestingly, we observed that 95 (6.1%) G1b sequences presented the polymorphism (L31 M) associated with resistance to DCV/IDX719, while this mutant was detected in only 6 (0.5%) G1a sequences conferring resistance to DCV/LDV/IDX719/ELV (*p* < 0.0001), and that 28 (2.3%) G1a and none G1b isolates harbored RAP (M28V) to OMV (*p* < 0.0001, Table 1).

**Figure 2:**
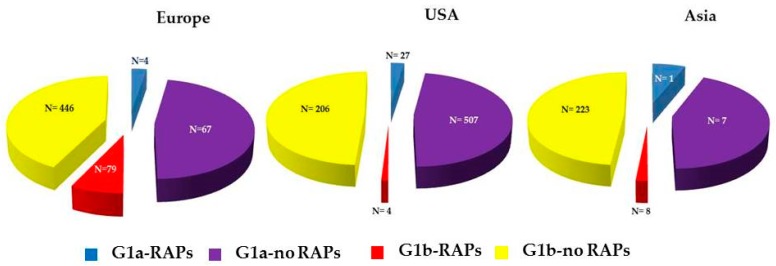
Frequency of RAPs according to geographic origin of HCV genotypes (G1a, G1b).

Concerning the geographical distribution of resistant variants, RAPs were detected in 83/596 (14%) European sequences, in 31/744 (4.2%) isolates from USA, and in 9/239 (3.7%) isolates from Asia (*p* < 0.0001). Sequences from Europe showed a higher frequency of RAPs compared to sequences from USA (*p* < 0.0001) and Asia (*p* < 0.0001). Frequency of RAPs was similar in sequences from USA and from Asia (*p* = 1), Figure 2. The frequency of RAPs was higher in G1b European isolates compared to G1a European isolates (15% *vs*. 5.6%, *p* = 0.029). The comparison between G1b and G1a in USA sequences group showed a trend towards significance (2% *vs.* 5%, *p* = 0.065).

The frequency of RAPs was similar in Europe and USA G1a isolates (5.6% *vs.* 5%, *p* = 0.775). On the contrary, the frequency of RAPs was higher in G1b European isolates compared to G1b isolates from USA (15% *vs.* 2%, *p* < 0.0001). The comparison among G1b sequences from Europe, USA, and Asia showed a higher frequency of RAPs in European isolates (*p* < 0.0001).

## 4. Discussion

NS5a inhibitors are characterized by their broad genotypic coverage and relatively low barriers to resistance. In this context, it is possible that some genotypes are naturally more prone to harbor lower susceptibility to anti-HCV inhibitors than others. The impact of baseline polymorphisms associated with loss of susceptibility to NS5a inhibitors was evaluated in phase 2 and phase 3 completed trials showing that the most common resistant mutations in G1a patients who did not achieve SVR were located at positions M28, Q30, L31, H58, and Y93, while in G1b were located at position L31 and Y93, suggesting that baseline polymorphisms at these sites may reduce the barrier to resistance and influence virologic outcome [14]. Two recent studies performed in Sweden [15] and Italy [16] in small groups of patients, found 1.6% and 12.5% of RAPs, respectively, in G1a clinical strains of individuals who have never been exposed to a drug. Additionally, the study from Italy found 53.3% of RAPs in G1b-infected patients. The high frequency of G1b resistance in this study is consequent to the fact that was considered in G1b, L28V, and Q54H aa substitutions, which are not shown to be relevant in clinical trials.

By sequence analysis of NS5a sequences retrieved from the Los Alamos database including isolates from all around the world, we found that HCV G1 had 7.4% of RAPs and that, comparing G1a with G1b, HCV-G1b had a higher frequency of polymorphisms (but not as high as in the study from Italy) at sites associated with resistance to NS5a inhibitors compared to HCV-G1a (*p* < 0.0001). The fold change, considering single mutation, was consistently higher for HCV-G1a compared to HCV-G1b. Therefore, the resistance levels of HCV-G1b single variants were relatively low compared to those of HCV G1a variants. In this context, *in vitro* studies on DCV have highlighted that the loss of susceptibility in G1a by the selection of resistance mutations, ranging from 233- to 3350-fold, whereas mutations at similar positions only resulted in a 3–28-fold loss in susceptibility to genotype 1b [17,18,19]. However, in DCV experienced patients, the most commonly observed aa changes in G1b infected patients who did not achieve SVR were low-level resistance substitutions L31M/V and Y93H [10].

A similar resistance level was present in G1b/OMV experienced patients exhibiting Y93H aa change, while, in G1b/LDV patients who did not achieve SVR, a high-level resistance Y93H aa change was found. Therefore, it is possible that clinically relevant resistance is not invariably associated with the resistance level (expressed by mean fold change) observed *in vitro* [10].

Additionally, in the present study, the single genetic polymorphism at critical positions (L31 and Y93) was detected in 6.1% and 2.4% HCV G1b natural strains retrieved from the Los Alamos database, suggesting that these polymorphisms could be involved in the early emergence of a double mutant with high-level resistance under drug pressure, also in G1b. In this context, a very recent study on grazoprevir-elbasvir combination therapy [20] showed that one/18 1b infected patients with Y93H aa substitution at baseline relapsed HCV infection with a double mutant.

In regard to cross-resistance to different NS5a inhibitors, we showed that, of 69 G1a sequences with RAPs, 31 had cross-resistance: 13 isolates were resistant to DCV/LDV/IDX719/ELV, 3 showed resistance to LDV/OMV/ELV, and the remaining 15 sequences had cross-resistance to all these compounds. In G1b, of 137 mutants, 37 showed cross-resistance to all NS5a inhibitors considered: DCV, LDV, OMV, IDX719, and ELV. The comparison between G1a and G1b mutated sequences indicated that cross-resistance pattern was a more common event in G1b than in G1a isolates, probably suggesting a lower genetic barrier of first-generation and second-wave NS5a inhibitors against G1b.

Second-wave NS5a inhibitors may display less or improved potency *in vitro* against resistant variants selected by DCV. Among the most frequent mutations detected in the present study, the Y93H conferring low-level resistance (12–93-fold) to DCV, OMV, IDX719, and ELV confers >900-fold resistance to LDV in G1b and was found in 37 G1b sequences. M28V substitution that was found in 28 G1a sequences confers resistance (>50-fold) to OMV but not to DCV/LDV/IDX719/ELV. L31M substitution conferring low-level resistance (3–3.6-fold) to DCV and IDX719 in G1b confers high-level resistance (105–554-fold) to DCV/LDV/IDX719 and low-level resistance (10-fold) to ELV in G1a. M28V substitution that was found in 28 G1a sequences confers resistance (>50-fold) to OMV but not to DCV/LDV/IDX719/ELV.

The unique double mutant of G1b we detected confers high-level resistance to DCV, while the resistance level to OMV/LDV/IDX719/ELV is unknown.

Concerning the geographic distribution of resistant variants, our analysis showed that RAPs were prevalently detected in sequences from Europe and in particular in G1b isolates, indicating a different NS5a resistant profile according to geographic origin of subtype 1b.

## 5. Conclusions

In summary, this study indicates that baseline polymorphisms associated with resistance to different NS5a inhibitors is not an infrequent event. The precise clinical implication of this finding remains to be established. However, it could be important to determine the mutational pattern of the NS5a domain1 according to different HCV subtypes and their geographic origin, especially in difficult-to-cure patients for whom new treatment strategies could involve complex association regimens.

## Figures and Tables

**Table 1:** Mean fold change in resistance compared to wild-type replicon of RAPs detected in G1a and G1b sequences retrieved from the Los Alamos HCV database and derived from NS5a inhibitors-naive patients.

RAPs	N ° of Isolates (%)	DCV * FC	LDV ° FC	OMV ^ FC	IDX719 °° FC	ELV ** FC
(1a)						
M28T	4 (0.3)	205	61	8695	150	15
M28V	28 (2.3)			58		
Q30H	7 (0.6)	435	183		24	6
Q30R	4 (0.3)	365	632	800	10	16
L31M	6 (0.5)	105	554		310	10
H58D	3 (0.2)		1127	243		6
Y93N	2 (0.2)	14,100	>14,706	66,740	14,000	929
Y93C	3 (0.2)	555	1602	1675	40	11
Y93H	2 (0.2)	1600	1677	41,303	4400	220
M28V+Q30H ^&^	2 (0.2)					
M28V+Q30R ^&^	1 (0.1)					
M28V+Y93C ^&^	1 (0.1)					
Q30H+L31M ^&^	1 (0.1)					
Q30H+Y93H ^&^	3 (0.2)					
L31M+Y93C ^&^	2 (0.2)					
(1b)						
L31M	95 (6.1)	3			3.6	
L31F	1 (0.06)			10	4	15
Y93H	37 (2.4)	12	994	77	93	17
L31M+Y93H	4 (0.3)	4227				

* DCV = daclatasvir; **°** LDV = ledipasvir; ^ OMV = ombitasvir; **°°** IDX719 = samatasvir, ** ELV = elbasvir. FC = mean fold change in resistance compared to wild-type replicon. Empty cells indicate no data available from patients who experienced treatment failure. ^&^ = Double resistant mutants that harbored 2 aa substitutions not detected in clinical trials.
